# Morphology and ploidy level determination of *Pteris vittata* callus during induction and regeneration

**DOI:** 10.1186/s12896-014-0096-6

**Published:** 2014-11-18

**Authors:** Blake L Joyce, Shigetoshi Eda, John Dunlap, C Neal Stewart

**Affiliations:** Department of Plant Sciences, The University of Tennessee, Knoxville, Tennessee 37996 USA; Center for Wildlife Health, Department of Forestry, Wildlife and Fisheries, the University of Tennessee, Knoxville, Tennessee 37996 USA; Advanced Microscopy and Imaging Center, The University of Tennessee, Knoxville, TN 37996 USA

**Keywords:** Extracellular matrix, Phytoremediation, Chinese brake fern, *Pteris vittata*, Scanning electron microscopy, Tissue culture and transformation, Transmission electron microscopy

## Abstract

**Background:**

Morphological and ploidy changes of the arsenic hyperaccumulator, Chinese brake fern (*Pteris vittata*) callus tissue are described here to provide insight into fern life cycle biology and for possible biotechnology applications. *Pteris vittata* callus was studied using transmission and scanning electron microscopy, and flow cytometry.

**Results:**

Callus induction occurred both in light and dark culture conditions from prothallus tissues, whereas rhizoid formation occurred only in dark culture conditions. Callus tissues contained two types of cells: one actively dividing and the other containing a single large vacuole undergoing exocytosis. Sporophytes regenerated from callus asynchronously form clusters of cells in a manner apparently analogous to direct organogenesis. Extracellular matrices were observed in actively-growing callus and at the base of regenerating sporophytes. Callus tissue nuclei were found to be primarily diploid at induction and throughout maintenance of cultures indicating that callus cell fate is determined at induction, which closely follows apogamous sporophyte development. Presence of a dense extracellular matrix in conjunction with sporophyte development suggests a link between the suspensor-like activity of the embryonic foot during normal fern embryo development and the suspected functions of extracellular matrices in angiosperms.

**Conclusions:**

Further investigation could lead to a better understanding of genes involved in *P. vittata* embryo development and apogamous sporophyte development. The methodology could be useful for in vitro propagation of rare and valuable fern germplasm.

**Electronic supplementary material:**

The online version of this article (doi:10.1186/s12896-014-0096-6) contains supplementary material, which is available to authorized users.

## Background

The Chinese brake fern (*Pteris vittata*) is an arsenic hyperaccumulator capable of storing arsenic in leaves up to 2.3% dry weight [1]. As such, this species has been a focus for use in phytoremediation. A fundamental problem in developing tissue culture and transformation systems *de novo* is ill defined tissue stages; i.e., distinguishing proliferating tissues as callus, somatic embryos or other differentiated tissue. Scanning electron microscopy (SEM), transmission electron microscopy (TEM), and histology have been used to characterize tissue culture systems [2-6] as well as the fern life cycle [7-12]. Even though *P. vittata* transformation has recently been accomplished using spores as transformation targets [13], there remains interest in developing tissue culture systems in this and other fern species. In this respect, fern tissue culture has lagged behind that of many other plants. There have been several reports of callus induction from *P. vittata* tissue [14-18]. Callus has been induced from fern explants ranging from prothalli, croziers, rhizomes, and even pinnae strips. These reports focus on culture conditions and nutritional requirements necessary for tissue culture, but do not fully characterize the events occurring during development, maintenance, and differentiation of callus.

Fern tissue culture and transformation technologies would yield possibilities for new areas of research investigating fern physiology and developmental biology through study of prothalli genes using overexpression and knockout analysis. Research investigating fern genomics and proteomics could yield new tools for molecular biology including promoters and genes responsible for arsenic accumulation pathways that can be transferred to other organisms to confer value-added traits for academic research and industrial applications.

The objective of our study was to characterize *P. vittata* callus induction, maintenance, and the subsequent sporophytes recovered from calli through use of transmission and scanning electron microscopy and flow cytometry.

## Methods

### Environmental conditions and tissue culture

Spores were harvested from mature P. vittata (courtesy of Edenspace Systems Corporation) fronds, using a straight-edged razor blade and collected into 1.7 ml centrifuge tubes (Denville, Metuchen, NJ, USA). A volume of 0.1 mL of spores was surface sterilized by sequential washes of 10% dilution of commercial chlorine bleach (5.25% sodium hypochlorite, Fisher Scientific, Pittsburgh, PA, USA) and 70% ethanol. They were then rinsed three times with sterile water and plated onto medium which contained half-strength Murashige-Skoog salts [19], B5 vitamins [20], 2.0% sucrose, and solidified with 0.2% Gelzan (Sigma) hereto referred to as 0.5MS20gS. Plates of sterilized spores were placed in lighted conditions to germinate. After about four weeks prothalli formed and were transferred onto callus induction medium which consisted of 0.5MS20gS supplemented with 0.5 mg L^***−***1^ gibberellic acid (GA_3_) and 0.5 mg L-1 6-benzylaminopurine (BA): Yang medium [21].

Light and dark environmental conditions were used for tissue culture. The light culture environment consisted of 16 hours of light at 79 μmol m^***−***2^ s^***−***1^ and 8 hours of darkness. The dark environment consisted of 24 hours of darkness. Both environments had a constant temperature of 25***°***C.

Fern tissue culture plates were then placed in either light or dark culture conditions. Every two weeks the prothalli were subcultured until they formed calli. Calli were excised and subcultured and maintained on the same medium in the same conditions or on liquid callus induction medium lacking Gelrite gellan gum in dark conditions.

To induce differentiation, calli were moved to medium containing half-strength MS salts supplemented with 6.0% maltose (Fisher Scientific), 0.5% activated charcoal (Sigma-Aldrich, St. Louis, MO, USA), and solidified with 0.2% Gelrite gellan gum. The calli were placed in lighted conditions and subcultured every two weeks until sporophytes were regenerated.

### Fixation of tissues

Fifteen samples from each stage of tissue culture were randomly selected and put into 3% glutaraldehyde in 0.1 M cacodylate buffer for 90 min at room temperature. They were then rinsed three times in the cacodylate buffer and transferred to 2% OsO_4_ in 0.1 M buffer for 90 min [22]. The samples were then dehydrated in a graded acetone series (25%, 50%, 75%, 95%, 100%, and dry 100%) for 30 min at each step. The samples were then divided into two groups according to preparation needed for the scanning or transmission electron microscope.

### Scanning electron microscopy

Samples were prepared using the critical-point drying method with carbon dioxide. Each tissue sample was affixed to two-sided carbon tape and then coated with 50 nm of gold. At least five samples were viewed from each stage of tissue culture and tissue culture condition.

### Transmission electron microscopy

Samples were transferred into a 1:3 acetone/Spurr mixture overnight. The samples were then moved to a 3: 1 acetone/Spurr mixture for 4 h with the beaker lid on and 4 h with the beaker lid removed. The samples were then placed in 100% Spurr’s resin and left overnight. Individual callus pieces were put into molds with fresh resin and placed in a 68°C oven for 24 h polymerize. At least three blocks for each condition were randomly selected and trimmed for thin-sectioning with glass knives on a microtome. Sections were collected on copper grids coated with glue. All grids were post-stained with uranyl acetate in 50% methanol for 30 min, and washed three times by 30 dips into distilled water. They were then stained in lead citrate for 5 min, washed again and then loaded for viewing using a Zeiss Libra 200MC transmission electron microscope.

### Flow cytometry

*P. vittata* callus, frond, and prothallus tissues were collected and weighed for nuclei extraction. For each preparation, approximately 0.1 g of each sampled tissue, in triplicate, was chopped in Otto buffer I using straight-edge razor blades [23]. Prothallus and callus controls were chopped independently, whereas the prothallus and callus tissues for genome size estimation comparison were co-chopped. After chopping, the suspension of each replicate was filtered by pipetting into a 5 ml polystyrene round-bottom tube with a 35 μm cell strainer cap (BD Falcon). Propidium iodide (50 μg ml^**−**1^) was added to the filtrate of each replicate.

The nuclei stained with propidium iodide were analyzed using a LSR II flow cytometer (BD Biosciences, San Jose, CA, USA) with the photomultiplier tube voltages set at 410 eV for forward scatter channel, 229 eV for side scatter channel, and 639 eV for the FL2 channel. The flow cytometer was equipped with a coherent sapphire 20 mW solid state 488 nm laser for the FL2 channel. Fluorescence of the stained nuclei was detected by using 555DCLP dichroic mirror and 575/26 nm bandpass filter. The threshold was set at 30,000 on the FL2 channel to exclude unstained particles in the suspension. The flow rate was set to 1.0 μl/s. Data, obtained by counting 5000 particles for each sample, were analyzed using DiVa software (BD Biosciences, San Jose, CA, USA). Signals from cellular debris were gated out on a 2-D dot plot of forward scatter (FSC) and side scatter (SSC) (Additional file 1).

## Results

### Callus induction

**Figure 1:**
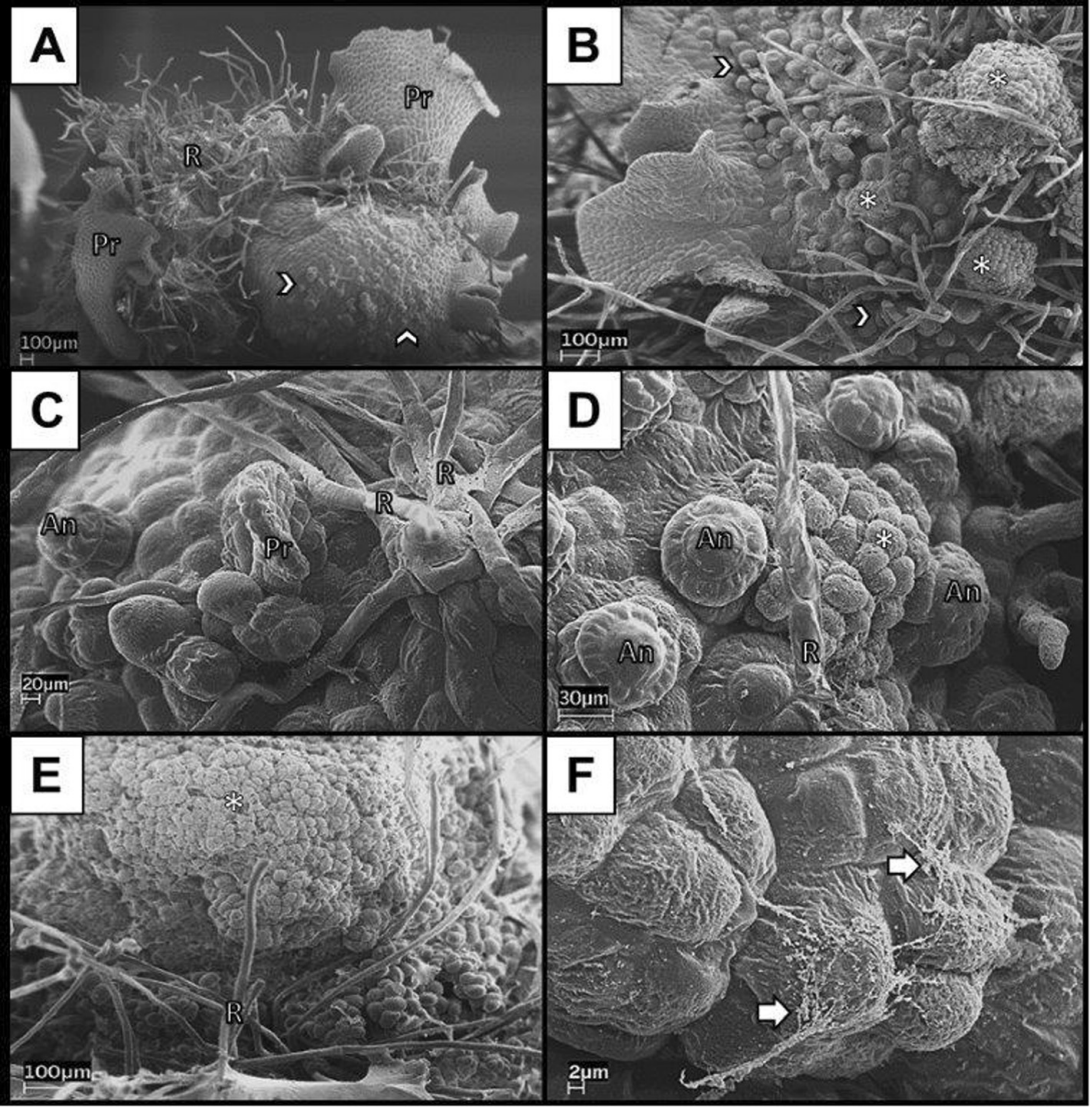
**Scanning electron microscopy of representative** ***Pteris vittata*** **prothalli grown under light culture conditions for six weeks (A, C, E) and dark conditions for six weeks (B, D, F). A)** Swollen prothallus with adventitious prothallus blades, rhizoids, and sex organs. Archegonia were observed, although the majority of sex organs were antheridia. **B)** Callus tissue forming on a swollen prothallus under dark conditions. Rhizoids were removed so that the prothallus surface could be viewed. **C)** Early formation of adventitious prothalli and rhizoids from antheridia-like structures. **D)** Callus tissue developed around antheridia cells. **E)** Formation of undifferentiated callus cells from the swollen prothallus. **F)** Fibrillar extracellular matrix covering dividing callus. Antheridia (An), callus (asterisk), extracellular matrix (arrow), groups of antheridia (chevron), rhizoids (R), adventitious prothallus blades (Pr).

Prothalli began to swell after four to six weeks of culture on callus induction medium in both light and dark conditions (Figure 1A and 1B). Adventitious prothalli, antheridia, archegonia, and rhizoids were present on the surface of the prothallus after six weeks of culture on callus induction medium in light and dark culture conditions (Figure 1A – D). Antheridia were the primary sex organ present on any surface of the prothalli, however a few archegonia were observed. In dark conditions, the prothalli formed thick mats of rhizoids which had to be removed for visualization with scanning electron microscopy. Rhizoids were observed elongating in a radial pattern from the basal cells of antheridia-like structures (Figure 1C and D). Undifferentiated cells forming callus were present on the surface of prothalli at eight weeks (Figure 1D). An extracellular matrix as well as fibrillar and granular structures were present on clusters of developing calli in both lighted and dark conditions (Figure 1E and 1F). Swelling gametophytes were excised and subcultured until the tissue evident in cultures appeared to consist exclusively of callus tissue (Additional file 1).

### Callus maintenance on semi-solid medium

Callus cultures grew rapidly on semi-solidified medium in both light and dark conditions. Callus differentiated to form sporophytes if the subculture interval exceeded two weeks (Additional file 1). On occasion, the explants died on the semi-solidified medium, but typically generated healthy callus tissue for subsequent subculturing.

**Figure 2:**
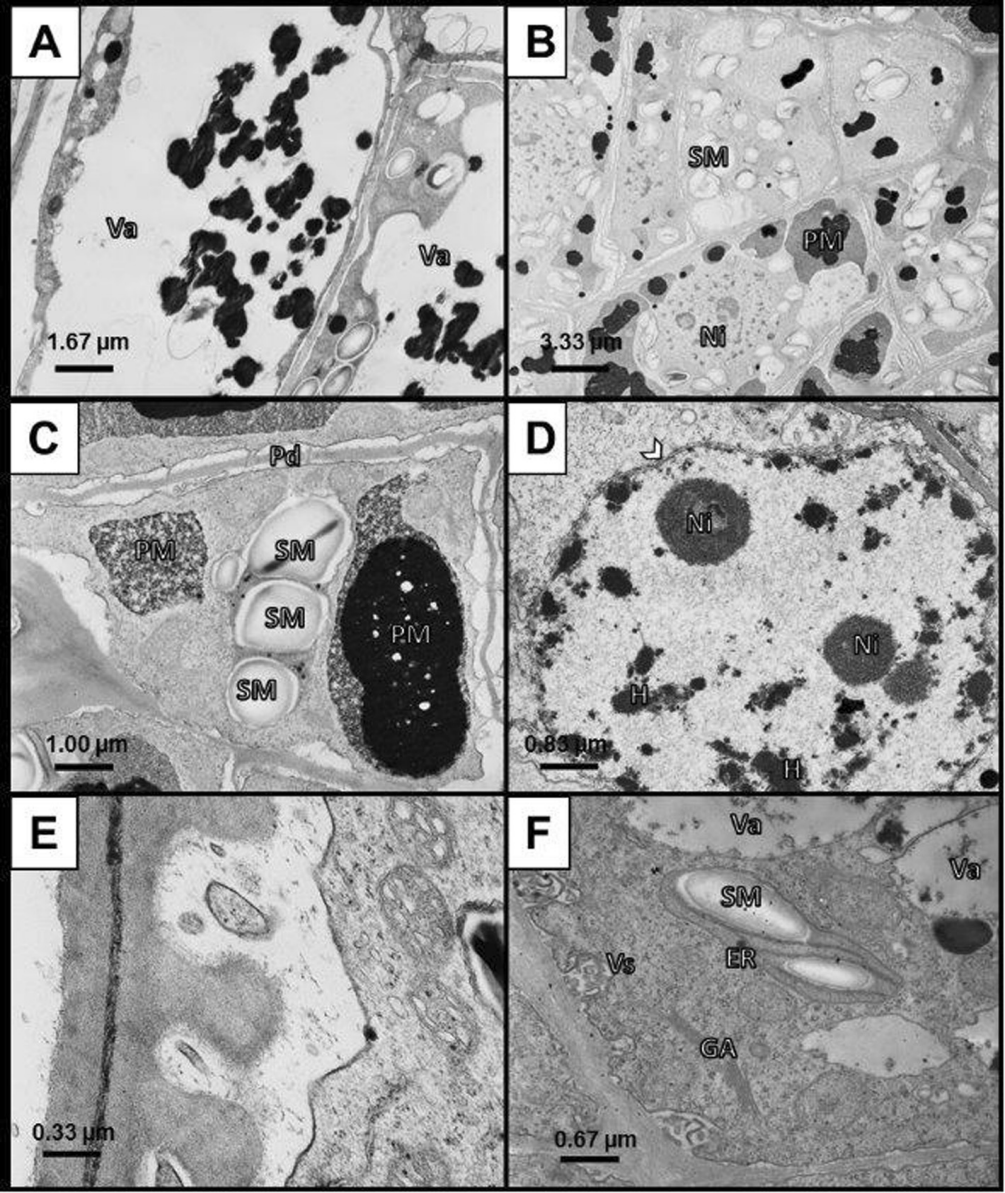
**Transmission electron microscopy of two cell types observed in** ***Pteris vittata*** **callus maintained on semi-solid callus induction medium. A)** The first cell type had a characteristically large vacuole with electron-dense vacuolar tannins and a thin layer of peripheral cytoplasm. **B)** The second cell type had a multitude of small vacuoles, nuclei with nucleoli, endoplasmic reticulum associated with starch granules, and Golgi apparati. **C)** Smaller cells of the second cell type contained more plasmodesmata, and dense cytoplasm with large amyloplasts and protein-filled vacuoles. **D)** Heterochromatin and two nucleoli were observed in all nuclei of the second cell type. **E)** Invaginations in the cell wall containing vesicles. **F)** Plastid surrounded by Golgi apparati and vesicles near the cell wall. Endoplasmic reticulum (ER), Golgi apparatus (GA), heterochromatin (H), nuclear envelope (NE), nucleoli (Ni), protein bodies (PM), plasmodesmata (Pd), starch granules (SM), vacuole (Va), vesicles (Vs).

Transmission electron microscopy of callus tissue showed the presence of two different cell types (Figure 2). Larger cells contained large vacuoles with electron dense regions, thin cytoplasm, and cell wall segments with few plasmodesmata (Figure 2A). Smaller cells each contained dense cytoplasm with large protein bodies and starch grains, small vacuoles, and a nucleus with two to several nucleoli in section and sparce heterochromatin (Figure 2B and 2D). Plasmodesmata are more abundant in the walls of the smaller cells than in the larger cells (Figure 2B).These cells also underwent cell wall biosynthesis as evidenced by nearby Golgi bodies (Figure 2F).

### Maintenance and regeneration of callus tissue

**Figure 3:**
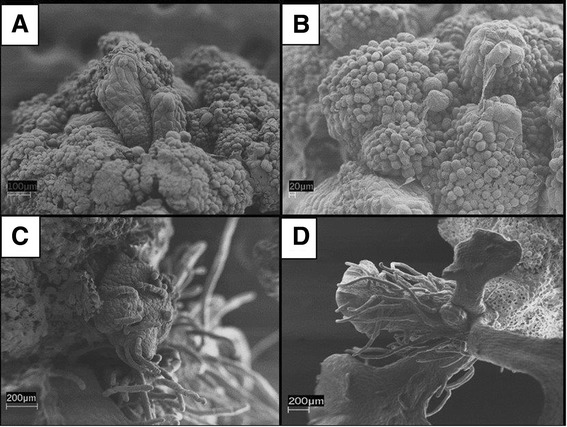
**Representative callus on Yang semi-solid medium for six weeks (A and B) and regenerating callus grown on differentiation medium under light culture conditions for four weeks (C) and eight weeks (D). A)** Spontaneous sporophytic growth occurred on callus during maintenance on callus induction medium. **B)** Typical round callus cells present on the callus before sporophytic growth. An extracellular matrix extends from cells. **C)** Asynchronous differentiation of sporophytes begins with clusters of cells from the callus surface when induced on differentiation medium. Differentiation of sporophytes was asynchronous. **D)** A crozier of a maturing sporophyte.

During callus maintenance, large differentiated terete structures formed from the center of the callus tissue (Figure 3A). Extracellular material seemed to be found on the surface of some callus (Figure 3B). Sporophyte differentiation occurred in clusters of cells that did not have the extracellular material (Figure 3C). Sporophyte regeneration was asynchronous and occurred across the surface of the callus tissue. After six weeks of regeneration, distinct mature sporophytes with croziers formed (Figure 3D). The cells at the base of sporophytes had thick, fibrillar extracellular matrix and were not spherical like the cells on the surface of the callus.

### Flow cytometry of prothallus and callus nuclei

**Figure 4:**
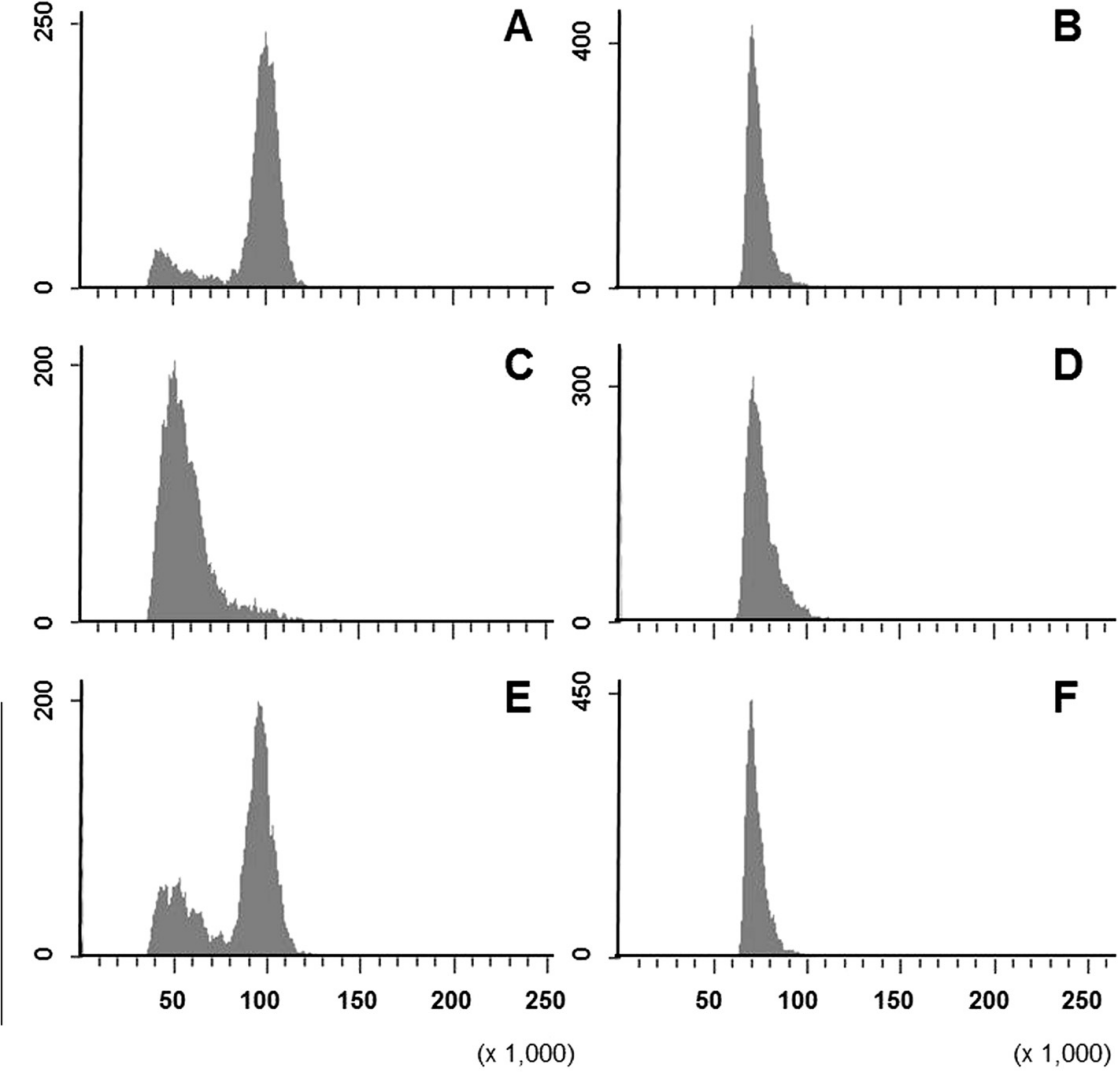
**Flow cytometry histograms of suspended** ***Pteris vittata*** **callus and prothallus nuclei stained with propidium iodide. A)** Isolation and staining of *P. vittata* callus control (2n) nuclei using PI-A calculation and **B)** PI-W. **C)** Isolation and staining of *P. vittata* prothallus control (1n) using PI-A calculation and **D)** PI-W. **E)** Dual isolation (co-chop) and staining of *P. vittata* prothallus (1n) nuclei exhibit one-half the fluorescence seen in callus nuclei (2n) using the PI-A. **F)** Co-chopping resulted prothallus and callus nuclei having the same peak in the PI-W histogram suggesting that the same particle size was counted for all events. Histograms are representative of three technical and biological replicates.

As seen in Figure 4, nuclei from callus tissue contained approximately twice the fluorescence intensity, and hence, approximately twice the DNA of the prothallus nuclei when quantified based on fluorescence pulse area (PI-A). Several small peaks were present at the cutoff limit of callus fluorescence (Figure 4A). These peaks were not continuous and did not have a mean at the expected gametophyte 1n level (50,000), which could represent numerous poorly-resolved haploid peaks (see also the Additional file 2). Alternatively, these peaks might represent cellular debris. Nonetheless, the data were reproducible (see also the Additional file 2). The ungated callus tissue flow diagrams did not have peaks, but rather a flat line near the 1n number (Additional file 2). The fluorescence pulse width (PI-W) formed a single peak at the nearly same value (approximately 75,000) in all the tested nuclei (Figure 4B,D, and F) demonstrating that the nuclei were approximately the same size.

## Discussion

Antheridia and archegonia were present across the entire gametophytic surface in both light and dark culture conditions even though in nature antheridia and archegonia are spatially separated. GA_3_ and ethylene interact to induce formation of antheridia in the fern *Anemia phyllitidis* [24], and ubiquitous production of sex organs has also been observed in other axenically cultured fern prothalli [9]. The presence of GA_3_ in the medium and production of ethylene in sealed plates could account for the ubiquitous presence of antheridia and rhizoids. Typically, the cap cells of antheridia open to release spermatozoa for fertilization, but no opened antheridia were observed on any prothalli in our experiments, which indicated that the fusion of sex cells did not occur prior to or during callus development.

SEM also revealed the presence of a fibrillar extracellular matrix on the outside the callus during callus induction and at the base of sporophytes during differentiation and development. Similar extracellular matrices have been observed in embryogenic and organogenic tissue cultures of flowering plants such as *Coffea arabica* [25], *Papaver somniferum* [4], *Brassica napus* [5], and *Actinidia deliciosa* [26]. Although the biological significance of extracellular matrices in tissue cultures is unknown, they have been considered as a biomarker for pre-embryogenic tissue [5] and involved in cell-cell adhesion and signaling during embryo development in some species [4,27]. The extracellular matrix, including collapsed cells at the base of the developing prothalli, in our cultures (Figure 3D) might be orthologous to the ‘foot’ development at the base of sporophytes on developing prothalli. The foot has been implicated in nutrient movement from gametophyte to sporophyte cells, which as also been compared to the suspensor of higher plant embryos [27].

Three types of calli were derived from *P. vittata* prothalli on basal medium the dark [18]. The type 2 callus described in Kato [18] had morphological similarities to our callus grown on Yang medium; both regenerated sporophytes. Kato [14] showed that including hormones such as indole-3-acetic acid (IAA) and GA_3_ promoted thickening of the prothallus pad and the formation of apogamous sporophytes. A mass of tissue derived from thickened prothalli of the fern *Pteridium* also resulted in apogamous sporophytes [12]. In this study, we observed the same thickening of prothalli exposed to BA and GA_3_ (Figure 1) during the formation of callus. From these calli, we recovered diploid sporophytic tissue, even though the callus was derived from haploid prothalli. We do not know by what mechanism this transition occurred. One explanation was that spontaneous apogamy occurred, since no antheridia or archegonia were observed during the experiments. While tissue culture-derived apogamy has not been shown in ferns before, it has been documented in the moss *Physcomitrium coorgense* [28]*.* Similar to our experiments the moss callus that produced apogamous sporophytes also accumulated large amounts of starch and contained an abundant number of plasmodesmata [29].

Another arsenic-hyperaccumulating fern, *Pteris cretica* [30], reproduces by obligate apogamy. Our *P. vittata* callus TEM images were congruent with observations of developing *P. cretica* egg cells with regards to the large number of free ribosomes, lipid bodies, and the many small vacuoles observed in each [31].

The production of sporophytes from *P. vittata* callus is an opportunity to investigate the genetic and morphological characteristics of prothalli during life cycle changes in pteridophytes. Histology of *P. vittata* rhizome callus has previously revealed distinct nodules of meristematic cells that gave rise to shoots, roots, or both, depending on concentrations of sugar and 2,4-D present in the medium [15]. In our experiments, *P. vittata* callus tissue was also comprised of cell clusters that differentiated into sporophytes. This type of callus most closely resembles embyrogenic callus of monocots and dicots, however, no secondary embyrogenesis deriving from single cells was observed in this work. Apogamous sporophyte production from gametophytes has been suggested as a strategy to continue fern life cycle either during dry environmental conditions that would not allow sperm motility or as a way for polyploid species to forgo mispaired chromosomes during meiosis. As regenerated sporophytes in this work produced spores and had no abnormal physiology they likely resulted from the natural apogamy life cycle during tissue culture [32].

Additionally, it is important to note that induced apogamy *in vitro* usually yields sporophytes with abnormal morphology [33], but the sporophytes derived from callus tissue in our experiments were indistinguishable from sporophytes grown on soil and produced viable spores that could germinate.

## Conclusion

Apogamous fern embryo development has been suggested as an early adaptation for colonizing dry land. Apogamous *P. vittata* callus could be further investigated to achieve a better understanding of genes and the physiology involved in *P. vittata* embryo development and apogamous sporophyte development. Additionally, the callus derived from *P. vittata* sporophytes was previously suggested as explants for transformation. However, callus does not seem to be suitable for use in the stable genetic transformation of *P. vittata*, and as such other targets for fern transformation are likely better.

## Additional files

Additional file 1:
**Development of callus from** ***Pteris vittata*** **gametophytes and regeneration of sporophytes from callus.**
Additional file 2:
**Flow cytometry dot blot and histograms showing gating on total events from extractions.**
