# Supplementary material for: Morphology and ploidy level determination of Pteris vittata callus during induction and regeneration
Source: BMC Biotechnol. 2014 Nov 18;14:96. doi: 10.1186/s12896-014-0096-6 (PMC4241211; doi:10.1186/s12896-014-0096-6)
Supplement: Additional file 1: — Development of callus from Pteris vittata gametophytes and regeneration of sporophytes from callus. [file 12896_2014_96_MOESM1_ESM.doc]

Additional file 1


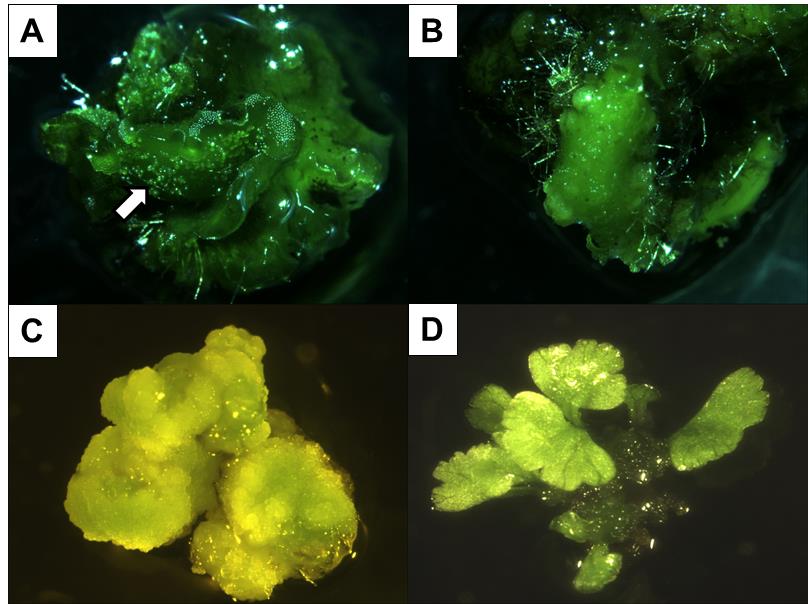


Additional file 1. Development of callus from *Pteris vittata* gametophytes and regeneration of sporophytes from callus. A) Gametophytes swell on Yang culture medium. Note presence of antheridia and archegonia on the outside of the swollen gametophyte (arrow). B) As the callus develops the gametophyte blade falls away. C) Subcultured callus on Yang medium. D) Initial development of sporophytic fronds from callus.
