# Supplementary material for: Morphology and ploidy level determination of Pteris vittata callus during induction and regeneration
Source: BMC Biotechnol. 2014 Nov 18;14:96. doi: 10.1186/s12896-014-0096-6 (PMC4241211; doi:10.1186/s12896-014-0096-6)
Supplement: Additional file 2: — Flow cytometry dot blot and histograms showing gating on total events from extractions. [file 12896_2014_96_MOESM2_ESM.docx]

Additional file 2

Flow cytometry dot blot and histograms showing gating on total events from extractions. A) Dot blot and gate on forward scatter (FSC) and side scatter (SSC) with *Pteris vittata* gametophyte nuclei extraction. B) Histogram of events inside the gate for *P. vittata* gametophyte nuclei extraction. C) Dot blot and gate on FSC and SSC with *P. vittata* callus nuclei extraction. D) Histogram of events inside the gate for *P. vittata* callus nuclei extraction. E) Dot blot and gate on FSC and SSC with *P. vittata* calli and prothallus nuclei co-chop extraction. F) Histogram of events inside the gate for *P. vittata* calli and prothallus co-chop extraction.


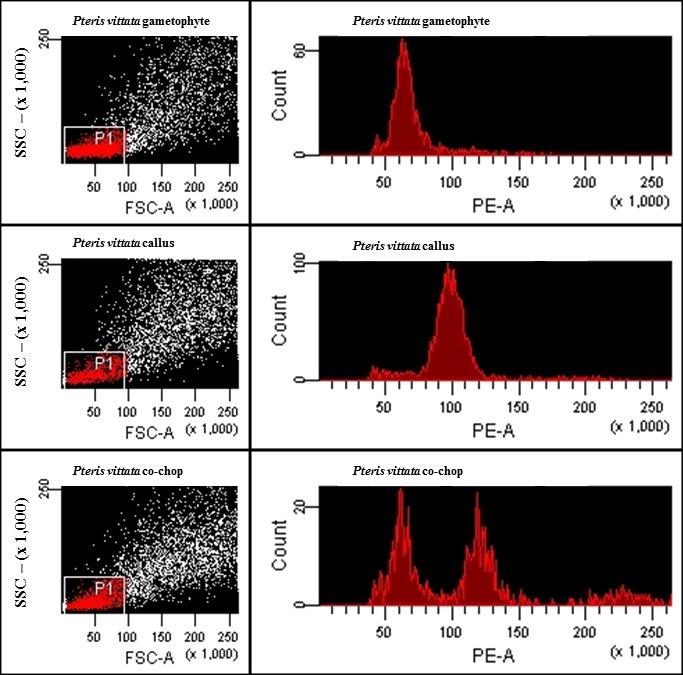


F

E

D

B

C

A
